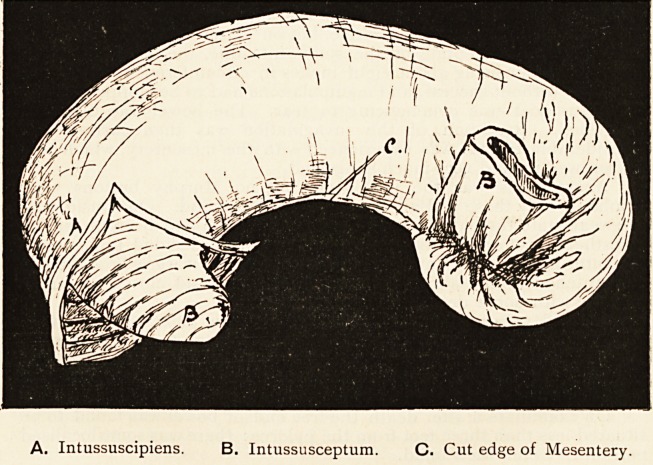# Irreducible Intussusception: With Notes on a Fatal Case

**Published:** 1899-12

**Authors:** G. L. Kerr Pringle

**Affiliations:** Surgeon to the Bridgwater Infirmary.


### IRREDUCIBLE INTUSSUSCEPTION:

### WITH NOTES ON A FATAL CASE.

BY

G. L. KERR PRINGLE, M.D. Ed.,

*Surgeon to the Bridgwater Infirmary.*

To obtain a correct knowledge of the best methods of procedure in surgery, it is necessary that we should acknowledge our failures as well as our successes, however distasteful that may be to us. Irreducible intussusception is one of the most desperate class of cases with which we have to deal in surgery, and all measures which we may adopt must be drastic.

Mr. D'Arcy Power,[1] in his Hunterian lectures on intussusception, considers that the outlook for irreducible cases treated by enterectomy is distinctly favourable. He says that Braun's[2]

[1] *Brit. M. J.*, 1897, i. 514; *Some Points in the Anatomy, Pathology, and Surgery of Intussusception*, 1898.

[2] *Verhandl. d. deutsch. Gesellsch. f. Chir.*, 1885, xiv. pt. 2, 491.

statistics, published in 1885, gave 1 recovery, and Rydygier,[1] in his addendum up to 1895, has 25 recoveries; to these may be added 10 more, Banks,[2] Pick,[3] Cripps, Heaton,[4] and others having had successful cases.[5] Thus we see that during the last fifteen years, owing to improved technic, etc., there has been a considerable reduction in the mortality.

At the present time five different methods have been suggested and carried out for the treatment of the irreducible bowel. These are:—i. To remove or excise the whole invagination, and to unite the two ends of the divided bowel in some manner or other. ii. To remove or excise the invagination and establish an artificial anus. iii. To leave the invagination and establish an artificial anus above it. iv. To short circuit the bowel and leave the invagination alone. v. To suture the entering piece of intestine to the ensheathing tube at its neck by a continuous suture, and then opening the ensheathing tube and extracting the intussusception, excising it within the sheath.

The first method is certainly the ideal one, but, unfortunately, patients are seldom in a condition to stand the extra shock caused by the time occupied in carrying out the anastomosis. Murphy's button considerably shortens the time, but it cannot well be used in all cases, such as the large intestine, as the presence of the appendices epiploicæ renders the two surfaces uneven and irregular. Of other methods, the end-to-end anastomosis by suture in the hands of an expert surgeon, backed up by assistants conversant with the surgeon's particular methods, is the most satisfactory. The temporary anastomosis forceps recently introduced by Laplace[6] of Philadelphia should considerably shorten the time occupied in suturing, for not only do they hold the parts well together, but they give the surgeon a command over the bowel which previously he has not had.

The second method appears to be the most feasible, and was

[1] *Verhandl. d. deutsch. Gesellsch. f. Chir.*, 1895, xxiv. pt. 2, 446.

[2] *Brit. M. J.*, 1896, ii. 1197. [3] *Quart. M. J.*, 1896–97, v. 121.

[4] *Brit. M. J.*, 1899, i. 958.

[5] Since above was written, Nicoll of Glasgow reports another successful case, *Brit. M. J.*, 1899, ii. 1094.

[6] *Ann. Surg.*, 1899, xxix. 297.

the one carried out in the subjoined case, but it is not applicable in those enteric cases where the obstruction is high up, as the patient will gradually sink from starvation.

Method No. 3 is the simplest, but here the patient is almost bound to have a resulting fæcal fistula, while the bowel below is in a state of gangrene till the intussusception is passed.

No. 4 likewise leaves the bowel to take care of itself, and the chances of peritonitis and gangrene are probable.

The fifth method, proposed by Rydygier[1] and carried out with modifications by Barker,[2] Greig Smith,[3] and Leszczynski,[4] has one great advantage, because there is not so much bowel removed, but this method is useless when the invaginated bowel is attached to the returning layer by adhesions for any great distance. It is also probable that leakage would occur, as a considerable portion of the ensheathing tube is occupied by the puckered-up mesentery.

The case which came under my notice is as follows :—

On August 10th I saw Mrs. W., in consultation with Dr. Wilberforce Thompson, who gave me the following history: The patient, a well-preserved woman of 50 years, had thirty years ago a local peritonitis which laid her up for a week. Six or seven months ago she had a sharp attack of colic which lasted some hours. She is inclined to be constipated; menstruation has not ceased, and at the present time she is unwell. Two mornings ago Dr. Thompson was sent for, the patient complaining of severe colicky pains in the region of the descending colon. She was given an opiate. Subsequently she vomited twice. She was kept under opium all day; bowels confined; temperature slightly raised; pulse, 70.

The following morning she was still suffering pain; bowels still confined. Opium continued. On seeing her, Dr. Thompson found the pain was slightly less, but the temperature had risen to 99.6°, and the pulse was 102. Pain is more in the region of the transverse colon. The abdomen is distended, and on each side below the level of the umbilicus there is a firm mass, dull on percussion; the rest of the abdomen is tympanitic. Examination *per rectum* revealed that the uterus was retroverted and the pelvic contents pressed down; the fundus of the uterus could not be felt bi-manually. A soap-and-water enema, with long tube, was given, which brought away four small scybala with blood-stained mucus. At 11 a.m. I saw her, and found her lying with knees drawn up, facial expression fair, temperature 99.6°, pulse 114; the abdomen distended and very painful to touch, a dull area just below the umbilicus on each side, flanks resonant; no vomiting, no hiccough. Patient passing wind by the mouth, but not by the anus.

[1] *Loc. cit.*, 439.

[2] *Lancet*, 1892, i. 79. [3] *Abdominal Surgery*, 5th Ed., 1896, vol. ii. p. 675.

[4] Quoted by Rydygier, *loc. cit.*

The symptoms being obscure and her environment unsatisfactory, she was removed to the Bridgwater Infirmary, where she was placed under ether. Nothing further could be made out on examining the abdomen, except that it was questionable whether the two masses felt were not connected. The hydrostatic douche was then tried with a long tube for some considerable time, but neither the tube nor the water would pass into the colon. The abdomen was then prepared and laparotomy performed, with the assistance of Dr. Thompson. An incision two inches in length was made to the right of the linea alba, which was subsequently enlarged to four inches, a large mass of bowel presented, coiled upon itself, having the appearance of a volvulus, as the bowel formed a complete circle, but on closer examination proved to be an intussusception of the small intestine, the sausage-shaped circular tumour being about eight inches in circumference: reduction was tried without success, and manipulations had to be stopped, as the subserous coat was commencing to tear. The bowel was dull and congested. Resection of the invagination was then carried out. Considerable trouble was experienced with the mesentery, which was all screwed up and very much congested.

Anastomosis was the next step, but no Murphy buttons were available, only those of Ball and Hey being to hand. On considering the weak state the patient was in and the time she had been under the anæsthetic (before the abdomen was opened), we decided to postpone the suturing to a later date, so both ends of the gut were brought out and stitched to the abdominal wall, and the wound closed as far as possible with some deep sutures.

She did very well for the first three or four days, but after that began to fail, the bowel acting constantly. Rectal feeding was most unsatisfactory, both suppositories and enemata being tried. She gradually got weaker, and died on the thirteenth day. At no time was her condition such as to allow of a second operation.

On examination after death the free end of bowel was found to be situated less than three feet from the pylorus; there was some localised peritonitis round the wound.

On examining the specimen subsequently, it was found that the valvulæ conniventes were large and numerous, showing that the invagination had taken place high up in the small intestine, and that the patient's chance of recovery was very doubtful.

The condition occurred about fifty hours before operation; adhesions had taken place, and the bowel was dull and much congested. To have carried out the method of Rydygier and Greig Smith would have been very difficult, if not impossible, as the entering piece of intestine was so much smaller and more puckered than the ensheathing portion.

If we had recognised at the time of operation that the obstruction was so near the stomach, immediate anastomosis would have been risked, but all the symptoms were against a high lesion. There had been no vomiting for two days, no hiccough; in fact, the signs and symptoms were by no means diagnostic of intussusception, for the bleeding *per rectum* was so slight that it might have been caused by the long tube, and, also, the patient was menstruating. The usual sausage-shaped tumour could not be felt on palpation, but in its place a double mass.

On considering the case, there is no doubt that had a

**A.** Intussuscipiens. **B.** Intussusceptum. **C.** Cut edge of Mesentery.

Murphy button been available it would have been the best method, on account of its rapidity.

Undoubtedly too much time was occupied in trying by irrigation to relieve the obstruction, but one is loath to operate if other means will avail.

---

## Figures and Tables

**A. B. C. f1:**